# Discussing sudden unexpected death in epilepsy: Are we empowering our patients? A questionnaire survey

**DOI:** 10.1177/2054270416654358

**Published:** 2016-09-01

**Authors:** S Keddie, H Angus-Leppan, T Parker, S Toescu, A Nash, O Adewunmi, RSN Liu

**Affiliations:** 1 Department of Neurology, National Hospital of Neurology and Neurosurgery, London WC1N 3BG, UK; 2 Department of Neurology, Royal Free Hospital, London NW3 2QG, UK; 3 Department of Neurology, Queen Square Hospital, London WC1N 3BG, UK; 4 Neurosciences, University College London, London, UK

**Keywords:** Sudden Unexpected Death in Epilepsy, communication, patients, risks in epilepsy

## Abstract

**Objective:**

To examine patient knowledge about sudden unexpected death in epilepsy (SUDEP) compared to other risks in epilepsy. To explore patients’ experiences surrounding SUDEP disclosure and opinions on how information should be delivered.

**Design:**

A cross-sectional questionnaire.

**Setting:**

Royal Free Hospital, London outpatient epilepsy clinics.

**Participants:**

New and follow-up patients attending epilepsy clinics at a London teaching hospital over six months. Patients identified as being at risk of suffering negative emotional or psychological consequences of SUDEP discussions were excluded.

**Main outcome measures:**

Patient knowledge about epilepsy risks; patient opinion regarding source, timing and delivery of SUDEP information; impact on health seeking behaviour.

**Results:**

Ninety-eight per cent of patients were aware of medication adherence, 84% of factors influencing seizure frequency, 78% of driving regulations, 50% of SUDEP and 38% of status epilepticus; 72% of patients felt that SUDEP information should be given to all patients. Preferences for timing of SUDEP discussions varied between those wanting information at diagnosis (40%) and those preferring to receive it after three clinic appointments (18%) to avoid information overload at the first consultation. Emotional responses (48% positive, 38% negative) predominated over measurable behavioural change following SUDEP discussions.

**Conclusions:**

Less than half the patients knew about SUDEP and status epilepticus. Although the majority of patients with epilepsy wish to be informed about SUDEP early on in their diagnosis, information must be delivered in a way that promotes patient knowledge and empowerment.

## Introduction

Sudden unexpected death in epilepsy (SUDEP) is the unexpected, witnessed or unwitnessed, death in patients with epilepsy, with or without evidence for a seizure, and excluding documented status epilepticus, drowning or trauma, with no toxicological or anatomic cause for death found on post-mortem.^1,2^ The incidence of SUDEP varies – it is estimated to be 0.09 to 1.2/1000 person-years in the general epilepsy population, and 9.3/1000 person-years in epilepsy surgery candidates.^3,4^ An increased SUDEP risk is associated with high-seizure frequency, the presence of generalised tonic-clonic seizures,^5^ epilepsy duration,^6^ and AED polytherapy.^5,6^ Strict adherence to antiepileptic drugs (AEDs) and optimal seizure control are the only widely recognised preventative measures.^7^

Whether clinicians should discuss SUDEP with all patients and their family is a controversial yet important question. Those in favour of a blanket disclosure policy assert that patients have a right to fully understand the risks of their condition to inform decision-making and treatment.^8^ Kroner et al.^9^ found in a study of 1300 patients that over three-quarters wished to be informed about SUDEP, irrespective of their risk.^9^ This is countered by a concern that patients may suffer negative psychological consequences as a result of receiving unsolicited information about a phenomenon they feel they have little control over.^10^ The 2012 National Institute of Health and Care Excellence (NICE) guidelines state that health professionals should give patients tailored information about SUDEP, discuss the patient’s individual SUDEP risk and measures to reduce this risk.^11^ However, there is a lack of clarity on how, when and by whom this information should be given.

Surveys in the United Kingdom, America and Canada demonstrate that a minority of clinicians discuss SUDEP with all patients with epilepsy (PWE).^10^ Only 31% of UK Neurologists discussed SUDEP risk with all or the majority of their patients, with the commonest reason for disclosure being in response to patient request.^12^ The main expressed reason for non-disclosure was a fear of causing patient distress.

The disparity between national guidance and current clinical practice has not been studied. Few studies have explored patient opinion in depth as a means to defining best practice regarding SUDEP disclosure. The primary objective of this study was to, therefore, describe patient opinions regarding when and how information on SUDEP and epilepsy safety should be delivered. Secondary objectives were to establish whether health seeking behaviour is influenced by knowledge about SUDEP, and in what way.

## Methods

This study was approved by the NHS Research Ethics Service at the Royal Free Hospital. Three consultant epilepsy specialists and an epilepsy specialist nurse recruited patients into the study. Prior to consent, patients were provided with an information sheet that is routinely available in clinic. This covered general epilepsy-related information such as driving and medication adherence as well as specific risks such as status epilepticus and SUDEP. Consenting patients agreed to either complete the questionnaire electronically or to return it by post. A telephone reminder was performed at one and three months.

Questions were asked to identify patients’ existing level of understanding about their condition and its management. We also gathered information on how patients were informed about SUDEP, when, where and how SUDEP discussions should be made, and the impact the information has on patients’ self-management. Anonymised results from the questionnaires were stored in an encrypted database and analysed by three independent clinicians.

### Participants and setting

Consenting adult (>18 years) patients with epilepsy attending the specialist epilepsy outpatient clinics at the Royal Free Hospital without intellectual disability were eligible for study. Fifty patients with variable seizure types and severity were included in the study over a six-month period. Both new and follow-up patients were included in the study to reflect a typical outpatient population. Patients whom consultants suspected as being at risk of psychological harm from participation were excluded from the study.

## Results

Fifteen per cent (2/13) of neurologists at the Royal Free Hospital in London surveyed discussed SUDEP with all PWE. SUDEP was most likely to be discussed with patients with intractable epilepsy (7/13) and poor medication adherence (9/13). Common reasons for non-disclosure were time constraints in clinic (4/13) and patients perceived to be at a low risk of SUDEP (6/13).

Fifty patients of 74 (68%) consented to the study (mean age 37.4 years; standard deviation (SD) 14.32; range 20–74, 24 women, 26 men).

From the 50 patients studied, there were 12 patients with bilateral convulsive seizures, 8 with focal dyscognitive seizures, 1 focal with motor components, 3 focal with subjective sensory components, 21 focal dyscognitive evolving to bilateral convulsive seizures, 2 absence, 1 myoclonic, 1 with mixed bilateral convulsive and absence seizures and 1 with mixed bilateral convulsive, myoclonus and absence seizures.

### Patients’ pre-existing epilepsy knowledge

Patients were asked “prior to this study being performed, were you aware of the following issues regarding your epilepsy?” Results are shown in Table 1. Only half surveyed reported understanding the term SUDEP (50%), and only 38% were aware of status epilepticus.

**Table 1.** Patients’ knowledge regarding epilepsy.

	Response (*n* = 50)		
Issue	Yes	No	Percentage ‘yes’ (%)
Importance of taking medication regularly	49	1	98
What to do when missing a dose of medication	37	13	74
Driving regulations with epilepsy	39	11	78
What can make seizure frequency worse	42	8	84
What people should do during a seizure	38	12	76
The benefit of wearing medical ID devices	28	22	56
What high risk activities to avoid	36	14	72
What status epilepticus is	19	31	38
What sudden unexpected death in epilepsy is	25	25	50

**Table 2. table2-2054270416654358:** Examples of patients’ responses to the question ‘Describe what you know about SUDEP’.

‘Small percentage of patients who die of unknown causes but attributed to the epilepsy’
‘A higher risk of unexplained death in people suffering from seizures – particularly uncontrolled seizures. Possibly related to the heart/abnormal heart rate’
‘I believe it is a rare condition that occurs in people with epilepsy where they just die suddenly for no specific reason. Cause of death is unknown’
‘A death caused by a lot of strong unexpected seizures, possible relating to other health problems’
‘The sudden death of a patient due to seizure which may be affected by the patient's management of epilepsy and its risk factors. Severity, type of epilepsy and circumstances play a role too’

### Patients’ understanding of what SUDEP means

Patients were asked the open question “please describe what you already know about SUDEP?” (Table 2). Of the 25 respondents with prior knowledge of SUDEP, patients commented that the condition was rare (*n* = 9), that the cause was unknown (*n* = 8), that risk factors influenced the likelihood of it occurring (*n* = 6) and that it occurred during a seizure (*n* = 6). Two patients were under the impression that it only occurred during sleep.

### Sources of SUDEP information

Of the 25 patients aware of SUDEP prior to the study, six (24%) had received information from multiple sources. Nine patients had received information from their hospital doctor or epilepsy nurse specialist (36%), others from patient information sheets (*n* = 2, 8%), the internet (*n* = 3, 12%) or newspapers (*n* = 1, 4%). Four patients did not respond (16%). No patients were informed about SUDEP by their GP.

### Do patients feel information about SUDEP should be given to all patients?

Thirty-six of the 50 participants felt information about SUDEP should be given to all patients (72%). Of the 14 participants that disagreed with this statement, reasons for not informing patients were young age (*n* = 4), old age (*n* = 1), unstable psychological state (*n* = 3), patients with less severe epilepsy and thus a lower risk of SUDEP (*n* = 3) and no comment (*n* = 3).

### When do patients feel they should receive SUDEP information?

A significant proportion of patients felt that patients should be made aware of the risk from the outset. Others felt that information delivered at the time of diagnosis might overwhelm patients who were still dealing with the repercussions of a new diagnosis of epilepsy. Some patients selected a combination of responses commenting that SUDEP discussions should occur on multiple rather than single occasions. Reponses presented in the free text box demonstrate the diversity of opinions expressed (Table 3).

**Table 3.** Patients’ responses to ‘When do you think this information should be given?’

Option	Number (*n*)	Percentage (%)	Details
At diagnosis	20	40	‘It's a scary thought but patients would need to be aware of the danger’. ‘As soon as possible without shocking the patient, depends on the patient’s perception of epilepsy’. ‘It’s best to get all information at diagnosis’.
Second visit	3	6	‘Being told by the epilepsy nurse was easiest for me after my second visit’
3 + visits	9	18	‘Epilepsy diagnosis can be quite a lot to take in. Hearing about SUDEP straight away is frightening. People need to know about it’. ‘I think it would be too much of a shock to be given this information when diagnosed. The second visit would be too soon as you are still coming to terms with what you are dealing with as a person diagnosed with epilepsy’.
Through request	4	8	‘At any stage that the patient or family member requests it’.
Multiple responses	10	20	‘Will vary according to the patient and their current condition, side effects of medication, amount and frequency of support they receive from family and friends etc. = A DIFFICULT BALANCE’. ‘It could be introduced at peer groups for people with epilepsy’. ‘When the doctor feels the patient has trust in them’.
No response	4	8

**Table 4.** Patients’ responses to “What do you feel is the best way to provide patients with information about SUDEP?”

Option	Number (*n*)	Percentage (%)	Details
By doctor in clinic	14	28	‘It feels better to be told frightening things in person, it gives you the chance to ask questions’. ‘The doctor can tell you whether you are high risk or not of SUDEP’. ‘It feels more comfortable, and appropriate, face to face with doctor in clinic’.
By specialist nurse	3	6	‘I was happy with the information coming from the nurse. I felt I could ask questions, as I wasn’t being rushed to leave’.
Info sheet/website link	4	8	‘I think having the information on the sheet, along with phone numbers of nurse and email address, gave it an appropriate context. If it had been specially mentioned at first, it would have been overemphasised and I would have worried’.
Doctor and info sheet	8	16	‘It feels better to be told frightening things in person and it is useful to have information to take away and think about later’. ‘Information sheets are good once you know about SUDEP’.
Doctor and nurse	9	18	‘Whoever is in regular contact with patient about their care’.
No preference	12	24	‘It depends on the individual. Personally, I like any information as long as it’s straight away’. ‘It’s best to ask the patient how they would like it themselves as everyone is different’.

### What do patients believe is the best way to receive information about SUDEP?

The majority of patients agreed that the doctor or specialist nurse should provide information about SUDEP in clinic (*n* = 34, 68%), suggesting that the sensitive nature of the information is best explained in person, possibly supplemented by an information sheet (Table 4).

### Does the provision of SUDEP information result in positive or negative consequences for patients?

Twenty-four patients (48%) said that SUDEP information resulted in positive consequences (increased awareness, appreciation of life and planning for the future). Nineteen (38%) reported it did the reverse (fear, sadness and anxiety) (Table 5). Seven patients (14%) did not respond to this question.

**Table 5.** Positive and negative consequences of SUDEP information.

Positive	Negative
‘Knowing more about your illness is always positive’.	‘The thought that I could die at any moment without explanation is somewhat distressing’.
‘It's been an education to my family and friends’.	‘It's frightening’.
‘Feel better for learning about it’	‘Being slightly more afraid for my safety’.
‘Prefer to be informed of all aspects of anything which is a part of something with which I live’	‘I found it quite depressing and in some ways wish I had never heard of it’.
‘It's good that it's available now. Doctors tend to tip toe around these issues’.	‘Makes me a little anxious – that fear of the unknown’.
‘Makes me think about what I want to happen in the future and look to help others’.	‘I found it quite stressful’.
‘It is life changing to hear the potential consequences. Knowing what could happen makes you appreciate life more. If this is my destined way to go then I would be very happy but I do wish for a long life!’	‘Unsure – can't do anything to prevent it’.
‘It has allowed me to explain to family and friends’.	‘At my age, it adds to list of worries about health’.
‘I know when I should and shouldn’t be driving – curtailed risky activities’.	
‘I am curious about my condition and willing to research and understand it’.	

## Discussion

When exploring patient knowledge about epilepsy management in general, medication adherence, DVLA driving regulations and seizure triggers were familiar to most patients. A significant proportion of patients lack knowledge about the most serious complications of epilepsy, namely SUDEP and status epilepticus. This may reflect reluctance on the part of the health professional to discuss difficult and emotive topics, particularly given the time constraints in clinic. It is surprising that relatively fewer patients were aware of the risk of status epilepticus despite its median incidence being 40 per 100,000 life years^5^ compared with that of SUDEP at 9 per 100,000 life years.^5^ The relatively lower proportion of patients recalling information on status epilepticus may represent recall bias, in that SUDEP may trigger a stronger emotional response; however, it emphasises the importance of putting individual risks into perspective.

In line with other studies,^9^ three-quarters of our PWE felt that SUDEP should be discussed with all patients. Patients aware of SUDEP appear to be well informed although a few believed that nothing could be done to prevent SUDEP and seemed unaware of the relative risks.

The most commonly expressed preference for receiving SUDEP information appeared to be at the point of diagnosis from the patients’ hospital doctor or specialist nurse. Patients commented that although SUDEP information might alarm patients, it was important for patients to be aware of potential dangers. Such sensitive information should be delivered face to face, allowing patients to ask questions and to receive an assessment of their individualised SUDEP risk. A proportion of patients felt that information delivered at an early stage could be frightening and overwhelming although studies have suggested that this initial anxiety about SUDEP may dissipate by three months.^14^ The marked variation in preferred timing of such information may reflect individual coping styles.^13^ With this in mind, clinicians may have to use their discretion to gauge patient readiness for such discussions.

With regard to the consequences of receiving SUDEP information, 48% reported benefits and 38% experienced negative consequences. Not all study patients were aware of SUDEP prior to the study. Learning about SUDEP via the participant information sheet rather than a face to face discussion may have contributed to those experiencing negative consequences. This is a potential source of bias. Emotional responses, both positive and negative, predominated over behavioural change such as medication adherence, risk taking activities and seizure management. This overriding emotional response following SUDEP disclosure was also observed in studies of young persons with epilepsy.^14,15^

Other studies have found that patients experience negative consequences on being informed about SUDEP (43% worrying a little, 23% a lot), albeit short-lived.^8^ Our own study showed that almost half of patients suffered negative consequences from SUDEP discussions and 2–7% patients and caregivers consistently state that they wish they had never been informed about SUDEP.^9,14,15^

### Future implications – empowering patients

This study highlights the disconnect between national guidance on SUDEP disclosure and current clinical practice.

Some patients react to information on SUDEP with a degree of fatalism, believing it to be a terminal event over which they have little control. Over half the patients in one study believed their actions would not influence whether or not SUDEP would occur.^9,16^ This finding raises some concerns. Despite studies purporting that the majority of PWE wish to be informed about SUDEP, it would appear that this knowledge is not being translated into steps to mitigate individual SUDEP risk. This is likely to reflect poor information provision. The manner in which risks of epilepsy, including SUDEP, are relayed to patients may be fundamental to whether patients alter their health-seeking behaviour.

Instead, SUDEP information should be ‘packaged’ with information such as the importance of drug adherence and avoidance of seizure triggers. This would improve patient knowledge and place an emphasis on improved self-management skills.^16^ One useful resource is a SUDEP and safety checklist mobile application (Epsmon) that both healthcare providers and PWE can use which improves awareness of safety, prompts SUDEP discussions and calculates individualised risk which may trigger early intervention.^17^ As demonstrated in other fields of medicine, emphasising positive steps that can modify risks has the potential to both empower and educate patients.

### Generalisability to other populations

This study was conducted in a specialist clinic, so the population may include more severe epilepsy cases than those managed in primary care, reducing its generalisability to patients with milder epilepsy. Future studies should recruit from both community and primary care settings.

## Conclusion

Most patients want to know about SUDEP at an early stage of their epilepsy. Despite this, discussions about SUDEP have not demonstrated a positive behavioural change in patients’ self-management. It is therefore suggested that healthcare providers package SUDEP information with other risks in epilepsy with an emphasis on the positive steps patients can take to prevent such a tragic event from occurring.[Table table2-2054270416654358]

## Declarations

### Guarantor

The lead author and guarantor of the study (RSNL) affirms that the manuscript is an honest, accurate, and transparent account of the study being reported, no important aspects of the study have been omitted, and there are no discrepancies from the study as planned.
